# Retinal neurovascular coupling dysfunction and plasma metabolomic features as biomarkers of Alzheimer’s disease: an integrated diagnostic model

**DOI:** 10.3389/fnins.2026.1892148

**Published:** 2026-08-14

**Authors:** Pan Xiao, Yunxia Wang, Jian Li, Wangjun Li

**Affiliations:** 1 Department of Ophthalmology, The Fourth Affiliated Hospital of Soochow University, Suzhou Dushu Lake Hospital, Suzhou, China; 2 Department of Neurology, Changshu No. 2 People’s Hospital, Affiliated Changshu Hospital of Nantong University, Changshu, China; 3 Department of Ophthalmology, Changshu No.2 People’s Hospital, Affiliated Changshu Hospital of Nantong University, Changshu, China

**Keywords:** Alzheimer’s disease, biomarkers, metabolomics, neurovascular coupling, optical coherence tomography angiography, retina

## Abstract

**Objective:**

To explore features associated with impaired retinal neurovascular coupling among individuals diagnosed with Alzheimer’s disease (AD) and its association with alterations in plasma metabolomics, and to establish an integrated diagnostic model for evaluating its possible significance regarding early-stage identification of AD.

**Methods:**

We prospectively enrolled 85 AD patients, 82 subjects with mild cognitive impairment (MCI), and 83 cognitively normal controls. Retinal vessel density, foveal avascular zone (FAZ) parameters, choriocapillaris flow deficits (CCFDs), ganglion cell layer thickness, and neurovascular coupling index (NVCI) were quantified using optical coherence tomography angiography (OCTA). Untargeted plasma metabolomics was performed via ultra-high-performance liquid chromatography–quadrupole time-of-flight mass spectrometry. Differential metabolites and pathways were identified, followed by correlation, regression, and receiver operating characteristic (ROC) analyses.

**Results:**

Compared with controls, the AD group showed reduced superficial vascular plexus perfusion density (−7.1%, *P* < 0.001), enlarged FAZ area (+16.1%, *P* < 0.001), increased CCFDs (+28.3%, *P* < 0.001), decreased ganglion cell layer thickness (−18.3%, *P* < 0.001), and lower NVCI (−12.7%, *P* < 0.001). Values in the MCI group were intermediate. In metabolomic profiling, AD patients had lower leucine (−17.0%, *P* < 0.001) and phosphatidylcholine PC(16:0/20:4) (−19.4%, *P* < 0.001), but higher ceramide d18:1/24:1 (+33.6%, *P* < 0.001) and ceramide/sphingomyelin ratio (+22.2%, *P* < 0.001). NVCI positively correlated with leucine (*r* = 0.205, *q* = 0.0011) and negatively with ceramide d18:1/24:1 (*r* = −0.139, *q* = 0.0280). An integrated model combining FAZ area, CCFDs, NVCI, leucine, ceramide d18:1/24:1, and PC(16:0/20:4) discriminated AD from controls with an AUC of 0.908 (95% CI: 0.865–0.952), 83.5% sensitivity, and 88.0% specificity. For predicting MCI-to-AD progression, the AUC was 0.744 (95% CI: 0.669-0.819), with 57.6% sensitivity and 85.4% specificity.

**Conclusion:**

Retinal neurovascular coupling dysfunction was significantly associated with systemic metabolic disturbances (branched-chain amino acid deficiency and ceramide accumulation) in patients with AD. The OCTA-metabolomics integrated model offers excellent diagnostic performance, supporting its potential for non-invasive early AD detection and risk assessment.

## Introduction

Alzheimer’s disease (AD) is the most common neurodegenerative disorder and the leading contributor to dementia globally, creating a considerable burden among affected populations, families, and healthcare systems. According to the latest epidemiological estimates, the global number of patients with AD is projected to increase from approximately 69 million in 2024 to 127 million by 2050, representing nearly a twofold rise (Alzheimers and Dementia, 2023; Gustavsson et al., 2023). Across the entire AD continuum, including the preclinical, transitional, and dementia-phase stages, the number of affected individuals worldwide has been estimated at nearly 416 million individuals, representing 22% among populations older than 50 years (Gustavsson et al., 2023). In 2024, healthcare expenditures and long-term care costs associated with AD reached approximately 360 billion US dollars and are projected to surpass 1 trillion US dollars before 2050 (Alzheimers and Dementia, 2023). This increasingly severe public health challenge has highlighted the urgent need to develop effective approaches for early identification and therapeutic intervention.

Pathological features of AD include extracellular accumulation of amyloid-β plaques, intracellular hyperphosphorylation of tau protein resulting in neurofibrillary tangles, as well as progressive neuronal loss and cerebral atrophy. Nevertheless, accumulating evidence has suggested that neurovascular dysfunction plays a crucial role in AD pathogenesis, potentially serving as one of the earliest reliable disease biomarkers (Chen et al., 2025). Disruption of blood–brain barrier integrity, abnormal regulation of cerebral blood flow, and defective amyloid clearance collectively constitute the core features of AD-associated neurovascular pathology (Divecha et al., 2025; Zhao et al., 2025). Disruption of the blood–brain barrier not only compromises amyloid-β clearance but also accelerates disease progression through the promotion of neuroinflammation, oxidative stress, and vascular endothelial injury (Beretti et al., 2025). The “two-hit” vascular hypothesis further proposes that cerebrovascular injury alone may be sufficient to initiate neuronal damage and neurodegeneration, while simultaneously facilitating amyloid accumulation within the brain (Wang et al., 2026).

At present, definitive diagnosis of AD primarily relies on cerebrospinal fluid biomarker analysis or amyloid/tau positron emission tomography imaging. However, these approaches are limited by their invasive nature, high cost, and restricted accessibility, rendering them unsuitable for large-scale early screening. Consequently, the identification of noninvasive, convenient, and economically feasible biomarkers has become a major focus in AD research.

As an extension of the central nervous system in the periphery, the retina shares the same embryological origin, neural tissue architecture, and vascular characteristics as the brain, and has therefore been regarded as a unique window for monitoring cerebral pathological alterations (Olivares Ordoñez et al., 2025). The retina and brain exhibit substantial similarities in neurovascular coupling mechanisms, autoregulatory blood flow function, and the morphological and physiological characteristics of the blood–retinal and blood–brain barriers (Shen, 2025). In recent years, rapid advances in high-resolution retinal imaging technologies, particularly optical coherence tomography and OCTA, have facilitated noninvasive observation of retinal microvascular circulation and neural tissue architecture at micrometer-level resolution (Gaier and Liao, 2026). Multiple investigations have revealed that AD patients present diverse retinal alterations, including thinning of the retinal nerve fiber layer, reduced ganglion cell layer thickness, diminished retinal vascular density, and FAZ enlargement (Nikparast et al., 2025). Close associations have been reported between these retinal vascular-structural changes and pathological events in cerebral tissue, indicating the potential applicability of retinal biomarkers for early-stage AD identification.

In addition to neurovascular alterations, systemic metabolic dysregulation has also been implicated in AD. Metabolomic investigations have revealed extensive metabolic abnormalities in patients with AD, involving multiple metabolic pathways linked to energy production, amino acid turnover, and lipid regulation. Disturbances in branched-chain amino acid metabolism and sphingolipid metabolic processes have attracted substantial interest. Evidence from large prospective investigations has indicated that reduced plasma branched-chain amino acid concentrations correlate with elevated risks of dementia and AD (Ikeuchi et al., 2022). Sphingolipid metabolism, particularly the imbalance between ceramides and sphingomyelins, has been identified as a promising therapeutic target for AD (Baloni et al., 2022; Uranbileg et al., 2024). Ceramides not only promote amyloid-β generation but also directly participate in neuronal apoptosis, neuroinflammation, and vascular endothelial dysfunction (Jazvinšcak Jembrek et al., 2015). Nevertheless, the intrinsic relationship between retinal neurovascular alterations and systemic metabolic dysregulation, as well as their synergistic contribution to the pathogenesis of AD, remains unclear and insufficiently elucidated. Therefore, the present study was designed to systematically evaluate the characteristics of retinal neurovascular coupling dysfunction in patients with AD through the integration of OCTA and plasma metabolomic analyses, to investigate the relationships linking retinal changes with plasma metabolic biomarkers, and to establish an integrated diagnostic model.

## Materials and methods

**FIGURE 1:**
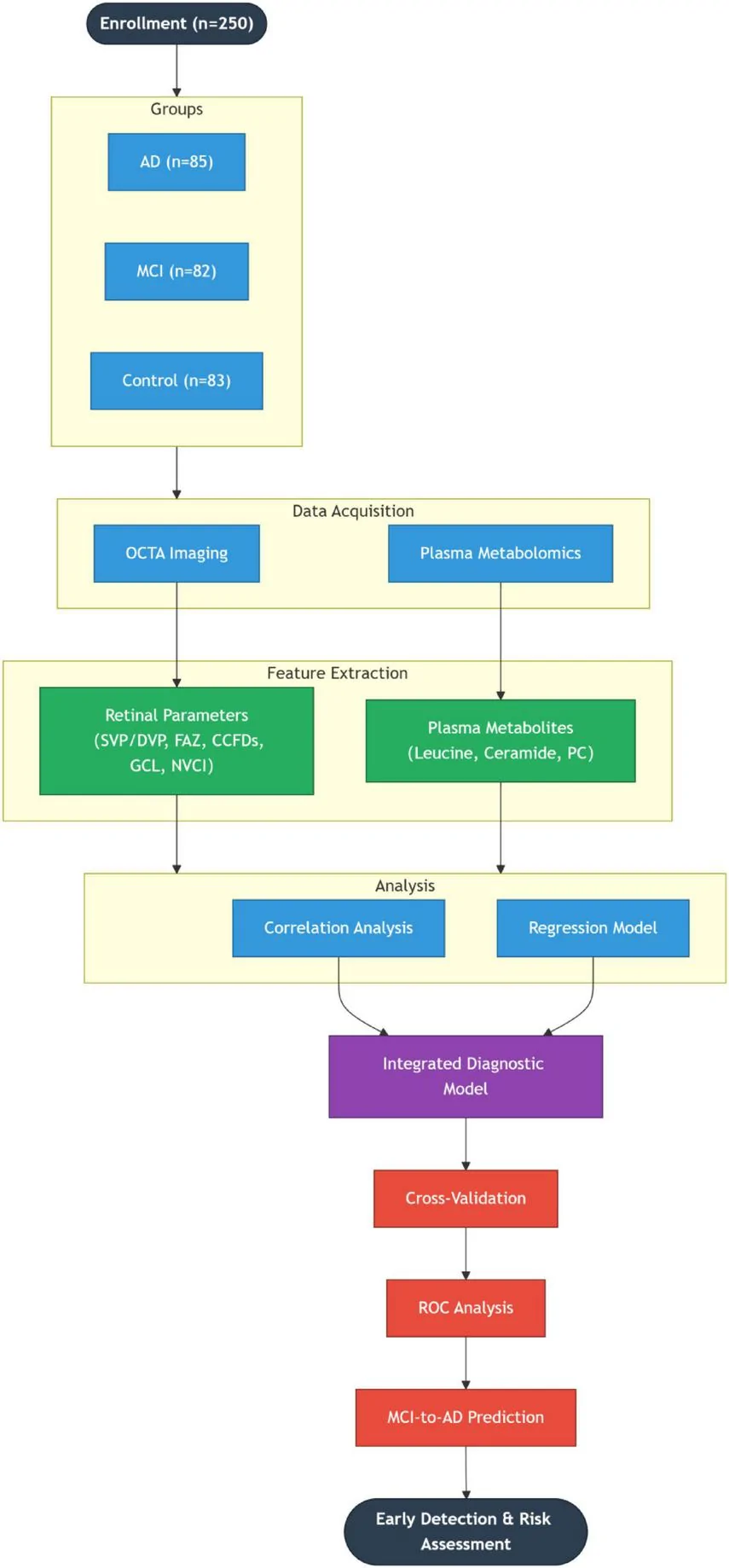
Study design and workflow.

Schematic overview of the study design and workflow was shown in Figure 1. A total of 250 participants were enrolled and classified into AD (*n* = 85), MCI (*n* = 82), and control (*n* = 83) groups. All participants underwent OCTA imaging and plasma metabolomic profiling. Retinal parameters (vascular density, FAZ area, CCFDs, GCL thickness, and NVCI) and plasma metabolites [leucine, ceramide d18:1/24:1, and PC(16:0/20:4)] were extracted. Correlation and regression analyses were performed to construct an integrated diagnostic model, which was validated by nested cross-validation and ROC analysis. The model’s predictive value for MCI-to-AD progression was also evaluated.

### Study population

The current study adopted a prospective case–control design. A total of 250 participants who attended the Department of Neurology, Department of Geriatrics, and Memory Clinic of our hospital between January 2023 and March 2025 were consecutively enrolled. This study was conducted in accordance with the Declaration of Helsinki. The study protocol was reviewed and approved by the Medical Ethics Committee of Changshu No.2 People’s Hospital (approval number: 2022-KYW-057). Based upon clinical diagnostic findings together with cognitive evaluation outcomes, participants underwent classification across three categories: AD cohort (85 subjects), MCI cohort (82 subjects), alongside cognitively healthy control cohort (83 subjects).

Eligibility criteria for the AD cohort included: age ranging between 60 and 85 years; meeting the 2018 updated AD diagnostic criteria proposed by the National Institute on Aging and the Alzheimer’s Association, which were based on clinical presentation, neuropsychological assessment, and structural MRI findings (hippocampal atrophy), without the use of amyloid or tau biomarkers; Mini-Mental State Examination (MMSE) scores between 18 and 24; Clinical Dementia Rating (CDR) scores between 0.5 and 1; and cranial magnetic resonance imaging findings consistent with characteristic AD-related alterations, including hippocampal atrophy and reduced medial temporal lobe volume. Participants in the MCI group were required to meet the Petersen diagnostic criteria for amnestic MCI, with MMSE scores between 24 and 27 and Montreal Cognitive Assessment (MoCA) scores between 18 and 25. In addition, subjective cognitive decline was required to be present, whereas activities of daily living were largely preserved.

The cognitively normal control cohort comprised healthy volunteers matched according to age together with sex, presenting MMSE scores exceeding 27, no subjective cognitive complaints, and neuropsychological test results within the normal range.

The exclusion criteria for all participants included the following: previous ophthalmic surgery or ocular trauma; ocular disorders affecting retinal structure or vasculature, including high myopia, glaucoma, macular degeneration, diabetic retinopathy, retinal vascular occlusion, or uveitis; refractive media opacity, such as significant cataract or vitreous hemorrhage, affecting image quality; other neurological disorders potentially influencing cognitive function, including Parkinson’s disease, vascular-origin dementia, frontotemporal dementia disorders, or normal-pressure hydrocephalus; uncontrolled systemic diseases, including diabetes mellitus, severe hypertension, or acute-stage cardiovascular and cerebrovascular diseases; history of major surgery or acute infection within the previous three months; long-term administration of medications potentially affecting metabolism, such as high-dose corticosteroids or immunosuppressive agents; and inability or unwillingness to cooperate with examinations. In all enrolled participants, best-corrected visual acuity remained above 0.5, whereas refractive error stayed within ± 6 diopters.

### Neuropsychological assessment

Comprehensive cognitive evaluations were performed on all participants by experienced neuropsychological specialists. The assessment battery included the MMSE for the evaluation of global cognitive function and the MoCA for multidomain cognitive assessment, including visuospatial ability, executive performance, naming capacity, memory function, attentional ability, linguistic competence, abstract thinking, alongside orientation capability. Dementia severity was determined using the CDR assessment. Moreover, episodic memory function was evaluated using the Auditory Verbal Learning Test. Semantic memory and naming performance were examined using the Boston Naming Test. Attention and executive performance were assessed using Trail Making Test Parts A/B. Processing speed was measured using the Digit Symbol Substitution Test. Daily functional competence was evaluated using the Activities of Daily Living Scale and the Instrumental Activities of Daily Living Scale. All assessment procedures were conducted in a standardized quiet environment, and the examination duration was limited to 60–90 min to minimize fatigue-related effects.

### OCTA examination

Retinal and choroidal imaging was performed using the PLEX Elite 9000 swept-source OCTA system (Carl Zeiss Meditec, Germany). The system was equipped with a 1,050-nm wavelength swept-source laser, featuring a scanning rate reaching 100,000 A-scans per second, axial resolution measuring 5 μm, alongside transverse resolution reaching 14 μm. Prior to image acquisition, all participants underwent dark adaptation for 10 min in a controlled dark-room environment. Examinations were subsequently conducted after full pharmacological pupil dilation. For each eye, images centered on the macular fovea were obtained using both 6 × 6 mm and 3 × 3 mm scanning protocols. Each scanning region was acquired three times, and the image with the optimal quality was selected for further analysis. Only images with a quality score exceeding 7 and a signal strength index greater than 60 were included in the final analysis.

Quantitative evaluation of retinal vascular indicators was completed using integrated vascular analysis software, enabling automated segmentation and measurement procedures. After automatic segmentation between the internal limiting membrane and the outer plexiform layer, retinal vasculature was classified into the superficial and deep vascular plexuses. The superficial vascular plexus was defined as the region extending from the internal limiting membrane to 10 μm beneath the inner plexiform layer. The deep vascular plexus was defined as the region spanning from 10 μm beneath the inner plexiform layer to 10 μm above the outer plexiform layer. Maximum entropy thresholding was applied for binarization of vascular images to separate vascular pixels from background pixels. The binarized images were subsequently processed using a skeletonization algorithm to extract vascular centerlines. Perfusion density was calculated as the percentage of vessel-occupied area relative to the total scanning region, whereas vessel length density referred to cumulative vascular skeleton length per unit area. Vascular fractal dimension was quantified using the box-counting method to evaluate the complexity and spatial filling characteristics of the vascular network.

Measurement of the FAZ was performed on superficial vascular plexus images obtained from the 3 × 3 mm scans. The built-in automated delineation function was used to identify the boundaries of the avascular region, followed by calculation of the FAZ area and perimeter. Circularity index was additionally calculated to evaluate morphological regularity. Analysis of CCFDs was conducted using en face images located 10–30 μm beneath Bruch’s membrane. A modified Phansalkar local thresholding algorithm was applied to identify hypoperfused regions, which were defined as areas with signal intensity lower than two standard deviations below the surrounding mean value. The percentage area occupied by flow deficits relative to the total region, along with the number and average area of flow deficits, were subsequently quantified. Retinal structural assessments included macular ganglion cell layer thickness and peripapillary retinal nerve fiber layer thickness. Automated segmentation algorithms integrated within the system were used to identify the boundaries of individual retinal layers. Ganglion cell layer thickness was measured within a 6-mm-diameter circular region centered on the fovea and was subsequently subdivided into the central region, inner ring, and outer ring, with each ring further subdivided into superior, inferior, nasal, and temporal quadrants. Retinal nerve fiber layer thickness was determined along a 3.46-mm-diameter circular scan surrounding the optic disc and was similarly divided into superior, inferior, nasal, and temporal quadrants. NVCI was defined as the ratio of ganglion cell layer thickness to superficial vascular plexus perfusion density within the corresponding region. While this index does not directly measure dynamic neurovascular coupling, it serves as a clinically accessible surrogate for the structural and functional matching between neuronal metabolic demand and local vascular supply. Previous studies have used similar indices to reflect the integrity of the neurovascular unit (Shen, 2025).

All images were independently analyzed by two experienced ophthalmologists. In cases of substantial discrepancy between measurements, consensus was achieved through discussion. The evaluators were blinded to the clinical diagnosis of participants in order to minimize potential bias. The mean values obtained from both eyes were used for statistical analysis. If image quality in one eye was inadequate, only data from the contralateral eye were included.

### Plasma metabolomic analysis

After completion of the ophthalmologic assessments, 5 mL of fasting venous blood was collected from each participant on the following morning after overnight fasting for more than 12 h. Blood specimens were placed in tubes containing ethylenediaminetetraacetic acid anticoagulant. Within 30 min after collection, Sample pretreatment was performed using a methanol-based protein precipitation method. Briefly, 400 μL of precooled methanol solution was added to 100 μL of plasma specimen, followed by vortex agitation for 1 min. Thereafter, incubation was performed at 4°C for 30 min to ensure complete protein precipitation, followed by centrifugation at 12,000 rpm for 15 min. Subsequently, 400 μL of supernatant was collected, filtered through a 0.22-μm membrane filter, and transferred to liquid chromatography sample vials maintained at 4°C before analysis. Quality-control specimens were prepared simultaneously by pooling equal volumes from all samples and processing them using the same protocol. One quality-control sample was inserted after every 10 study samples during analytical procedures to evaluate instrumental stability and reproducibility.

Metabolic profiling was performed using an ultra-high-performance liquid chromatography–quadrupole time-of-flight mass spectrometry platform. Chromatographic separation was performed on an ACQUITY UPLC BEH C18 column maintained at 45°C with a flow rate of 0.4 mL/min. Under positive ion mode, mobile phase A consisted of water supplemented with 0.1% formic acid, whereas mobile phase B consisted of acetonitrile supplemented with 0.1% formic acid. Under negative ion mode, mobile phase A consisted of 5 mmol/L ammonium acetate aqueous solution, whereas mobile phase B consisted of acetonitrile.

Gradient elution conditions were established as follows: mobile phase B remained at 5% throughout 0–2 min, linearly increased to 95% from 2 to 10 min, was maintained at 95% from 10 to 12 min, subsequently returned to 5% within 0.5 min, and equilibrated for an additional 3 min. The injection volume was 2 μL.

Mass spectrometric detection was performed using an electrospray ionization source, and data acquisition was conducted separately in positive and negative ion modes. Ion source settings were configured as follows: capillary voltage reaching 3,000 V; cone voltage, 40 V; source temperature, 120°C; desolvation gas temperature, 500°C; desolvation gas flow rate, 800 L/h; and cone gas flow rate, 50 L/h. The mass scanning range was set from 50 to 1,200 m/z, with an acquisition rate of 0.2 s per spectrum. Data acquisition occurred using MSE acquisition mode, enabling simultaneous collection of low-collision and high-collision energy spectra. Low collision energy remained fixed at 6 eV, whereas high collision energy increased progressively between 20 and 40 eV, generating abundant fragment ion information facilitating metabolite identification. Leucine enkephalin was used as the lock-mass correction compound at a concentration of 200 pg/μL and a flow rate of 10 μL/min, enabling real-time mass drift correction and ensuring mass accuracy.

Data processing was completed using Progenesis QI software enabling peak detection, peak alignment, normalization, alongside metabolite identification procedures. Retention time alignment tolerance was set at 0.2 min, and mass deviation was controlled within 5 ppm. Metabolite identification was conducted through comparison with databases including HMDB, METLIN, and LipidMaps, in combination with accurate mass measurements, isotope distribution, fragment ion patterns, and retention time information. Only metabolites with an identification confidence level of grade 2 or higher were included in subsequent analyses.

### Statistical analysis

Statistical analyses were completed using SPSS version 26.0 and R software version 4.2.0. Continuous variables were initially assessed for normality and homogeneity of variance. Normally distributed variables are presented as mean ± standard deviation, whereas intergroup differences were examined using one-way analysis of variance, followed by least significant difference *t*-tests for pairwise comparisons. Non-normally distributed variables are presented as median with interquartile range, whereas comparisons across groups were performed using the Kruskal–Wallis rank-sum test.

Categorical variables are presented as frequencies and percentages, whereas intergroup comparisons were performed using the chi-square test or Fisher’s exact test, as appropriate. Associations between retinal indicators and plasma metabolites were evaluated using Spearman correlation analysis. To correct for multiple comparisons, the Benjamini-Hochberg procedure was applied to control the false discovery rate (FDR), and an FDR-adjusted *P*-value (*q*-value) < 0.05 was considered statistically significant. For metabolomic data preprocessing, missing values were imputed using the k-nearest neighbor (k-NN) algorithm (*k* = 5). Data were normalized using the median fold-change method to reduce systematic variation between samples. Batch effects were adjusted using the ComBat method implemented in the sva package. Multivariate statistical evaluation of metabolomic datasets was performed using principal component analysis and partial least squares-discriminant analysis. Differential metabolites were screened using orthogonal partial least squares-discriminant analysis according to variable importance in projection values greater than 1 and adjusted *P <* 0.05. A multivariate stepwise regression model was established to identify independent predictive factors. ROC curve analysis was subsequently applied for diagnostic performance evaluation, and AUC, sensitivity, and specificity were calculated. The significance threshold α was set at 0.05, and two-tailed *P*-values below 0.05 were considered statistically significant.

## Results

### Comparison of general characteristics

A total of 250 participants were included in the current study, including 85 subjects in the AD cohort, 82 subjects in the MCI cohort, and 83 cognitively healthy controls. No statistically significant intergroup differences emerged regarding age, sex distribution, and years of education. However, statistically significant differences among groups were identified in MMSE scores, MoCA scores, and CDR scores. Detailed demographic alongside clinical features appear throughout Table 1. Cognitive function scores in both the AD and MCI groups were significantly lower than the other group. Cognitive impairment was markedly more severe in AD as well as MCI. No statistically significant intergroup differences emerged regarding baseline biochemical indicators, including blood pressure, fasting blood glucose, alongside blood lipid profiles, indicating satisfactory comparability between groups.

**TABLE 1:** Comparison of general characteristics and cognitive function among the three groups.

Variable	Control group (*n* = 83)	MCI group (*n* = 82)	AD group (*n* = 85)	*F*/χ ^2^-value	*P*-value
Age (years)	69.54 ± 5.41	70.21 ± 5.42	71.26 ± 6.10	1.961	0.143
Sex (male/female)	38/45	41/41	43/42	0.458	0.795
Years of education (years)	12.28 ± 3.10	11.60 ± 3.56	10.71 ± 3.57	1.591	0.206
Body mass index (kg/m^2^)	24.48 ± 2.66	23.82 ± 3.13	23.62 ± 3.87	0.947	0.389
Systolic blood pressure (mmHg)	127.96 ± 14.11	130.96 ± 13.50	130.00 ± 15.34	0.947	0.389
Diastolic blood pressure (mmHg)	78.48 ± 9.68	79.35 ± 9.12	80.13 ± 9.22	0.656	0.520
Fasting blood glucose (mmol/L)	5.22 ± 0.79	5.16 ± 0.67	5.27 ± 0.65	0.504	0.605
Total cholesterol (mmol/L)	4.81 ± 0.78	4.97 ± 0.98	4.94 ± 0.90	0.711	0.492
Triglycerides (mmol/L)	1.45 ± 0.53	1.49 ± 0.54	1.50 ± 0.57	0.226	0.798
MMSE score	27.75 ± 1.23	24.65 ± 3.79	19.26 ± 3.43	203.880	<0.001
MoCA score	24.35 ± 5.53	21.76 ± 5.34	18.98 ± 2.85	14.852	<0.001
CDR score	[0.00(0.00, 0.00)]	[0.00(0.00, 1.00)]	[1.00(0.00, 1.00)]	34.419	<0.001
AVLT delayed recall	9.72 ± 2.42	5.52 ± 1.92	2.44 ± 1.25	302.160	<0.001
Trail Making Test Part B (s)	65.34 ± 19.33	105.42 ± 45.86	108.91 ± 48.74	160.471	<0.001

### Comparison of retinal vascular parameters

OCTA analysis demonstrated significant alterations in retinal vascular parameters in the AD and MCI groups compared with the control group. Detailed results are presented in Table 2. Among retinal vascular parameters, perfusion densities of the superficial and deep vascular plexuses exhibited a gradual reduction from the control cohort to the MCI cohort and then to the AD cohort, with statistically significant differences observed among groups [superficial vascular plexus: (47.11 ± 8.57)%, (44.58 ± 8.15)%, and (43.78 ± 8.72)%, respectively, *F* = 3.507, *P* = 0.031; deep vascular plexus: (50.95 ± 8.84)%, (48.26 ± 8.52)%, and (47.83 ± 8.98)%, respectively, *F* = 3.091, *P* = 0.047]. In contrast, the FAZ area and CCFDs exhibited a stepwise increase across the three groups, and the intergroup differences were statistically significant [FAZ area: (0.31 ± 0.09) mm^2^, (0.34 ± 0.09) mm^2^, and (0.36 ± 0.08) mm^2^, respectively, *F* = 9.030, *P* < 0.001; CCFDs area: (12.34 ± 2.87)%, (13.76 ± 3.64)%, and (15.83 ± 4.52)%, respectively, *F* = 18.353, *P* < 0.001]. No statistically significant intergroup differences emerged regarding remaining vascular parameters (*P* > 0.05).

**TABLE 2:** Comparison of retinal vascular parameters among the three groups.

Parameter	Control group (*n* = 83)	MCI group (*n* = 82)	AD group (*n* = 85)	*F*-Value	*P*-value
Superficial vascular plexus					
Perfusion density (%)	47.11 ± 8.57	44.58 ± 8.15	43.78 ± 8.72	3.507	0.031
Vessel length density (mm/mm^2^)	18.84 ± 3.56	18.28 ± 3.89	17.64 ± 3.13	2.385	0.094
Vascular fractal dimension	1.65 ± 0.98	1.64 ± 0.91	1.62 ± 0.93	2.900	0.057
Deep vascular plexus					
Perfusion density (%)	50.95 ± 8.84	48.26 ± 8.52	47.83 ± 8.98	3.091	0.047
Vessel length density (mm/mm^2^)	21.05 ± 4.73	20.18 ± 4.04	19.57 ± 4.36	2.406	0.092
Vascular fractal dimension	1.70 ± 0.09	1.68 ± 0.09	1.67 ± 0.10	2.399	0.093
FAZ					
Area (mm^2^)	0.31 ± 0.09	0.34 ± 0.09	0.36 ± 0.08	9.030	<0.001
Perimeter (mm)	2.17 ± 0.51	2.28 ± 0.52	2.37 ± 0.59	2.588	0.077
Circularity index	0.77 ± 0.10	0.75 ± 0.09	0.74 ± 0.09	1.762	0.174
Choriocapillaris layer					
CCFDs area (%)	12.34 ± 2.87	13.76 ± 3.64	15.83 ± 4.52	18.353	<0.001
Number of flow deficits (counts/mm^2^)	9.53 ± 3.32	10.02 ± 4.15	11.01 ± 4.35	3.041	0.050
Mean flow deficit area (μm^2^)	1876.49 ± 588.48	1944.51 ± 557.50	2071.51 ± 528.49	2.611	0.075

### Comparison of retinal neural tissue structural parameters

Analysis of retinal neural tissue structure demonstrated significant reductions in ganglion cell layer thickness and retinal nerve fiber layer thickness in the AD and MCI cohorts relative to the control cohort. Comprehensive findings are shown in Table 3. Among retinal neural structural parameters, ganglion cell layer thickness in the central region, superior quadrant, and inferior quadrant showed a progressive decrease from the control cohort to the MCI cohort and then to the AD cohort, with statistically significant intergroup differences observed [central region: (16.85 ± 4.15) μm, (16.05 ± 3.65) μm, and (15.28 ± 4.15) μm, respectively, *F* = 3.225, *P* = 0.041; superior quadrant: (80.25 ± 10.25) μm, (77.75 ± 10.61) μm, and (76.33 ± 9.94) μm, respectively, *F* = 3.127, *P* = 0.046; inferior quadrant: (80.15 ± 13.85) μm, (76.85 ± 14.05) μm, and (74.85 ± 13.76) μm, respectively, *F* = 3.113, *P* = 0.046]. Similarly, both the overall NVCI and inner-ring NVCI exhibited a stepwise reduction across the three groups, with statistically significant differences identified among groups [overall NVCI: 1.65 ± 0.28, 1.57 ± 0.21, and 1.44 ± 0.25, respectively, *F* = 17.149, *P* < 0.001; inner-ring NVCI: 1.71 ± 0.50, 1.69 ± 0.44, and 1.54 ± 0.47, respectively, *F* = 3.110, *P* = 0.046]. No statistically significant intergroup differences were identified regarding remaining neural tissue parameters (*P* > 0.05).

**TABLE 3:** Comparison of retinal neural tissue structural parameters among the three groups.

Parameter	Control group (*n* = 83)	MCI group (*n* = 82)	AD group (*n* = 85)	*F-*value	*P-*value
Ganglion cell layer thickness (μm)					
Overall average	80.51 ± 10.20	78.25 ± 10.75	76.55 ± 10.85	2.940	0.055
Central region	16.85 ± 4.15	16.05 ± 3.65	15.28 ± 4.15	3.225	0.041
Inner ring	93.23 ± 13.08	91.65 ± 13.45	88.37 ± 12.79	3.012	0.051
Outer ring	73.05 ± 12.75	70.35 ± 12.85	68.45 ± 12.74	2.742	0.066
Superior quadrant	80.25 ± 10.25	77.75 ± 10.61	76.33 ± 9.94	3.127	0.046
Inferior quadrant	80.15 ± 13.85	76.85 ± 14.05	74.85 ± 13.76	3.113	0.046
Nasal quadrant	76.35 ± 16.85	73.45 ± 16.24	79.55 ± 16.35	2.861	0.059
Temporal quadrant	79.05 ± 15.25	75.95 ± 15.45	73.65 ± 15.55	2.591	0.077
Retinal nerve fiber layer thickness (μm)					
Overall average	94.05 ± 15.65	89.75 ± 15.95	88.42 ± 15.15	2.981	0.053
Superior quadrant	114.65 ± 19.35	110.25 ± 19.65	107.75 ± 19.25	2.711	0.068
Inferior quadrant	120.25 ± 23.75	114.55 ± 23.95	112.25 ± 23.65	2.510	0.083
Nasal quadrant	71.45 ± 12.95	69.85 ± 12.85	66.85 ± 11.75	2.927	0.055
Temporal quadrant	65.25 ± 11.86	63.35 ± 11.15	60.85 ± 12.25	2.957	0.054
NVCI					
Overall NVCI	1.65 ± 0.28	1.57 ± 0.21	1.44 ± 0.25	17.149	<0.001
Inner-ring NVCI	1.71 ± 0.50	1.69 ± 0.44	1.54 ± 0.47	3.110	0.046
Outer-ring NVCI	1.51 ± 0.50	1.40 ± 0.49	1.33 ± 0.47	2.778	0.064

### Results of plasma metabolomic analysis

Untargeted plasma metabolomic profiling detected a total of 8,746 metabolic feature peaks under both positive and negative ion modes. After quality control procedures and data preprocessing, 6,832 reliable features were retained for subsequent statistical analyses. Principal component analysis revealed an evident separation pattern among three groups regarding metabolic profiles, with the first and second principal components explaining 23.7 and 16.4% of the total variance, respectively. The partial least squares-discriminant analysis model yielded an R^2^Y value of 0.847 and a Q^2^ value of 0.723, indicating satisfactory model fitness and predictive performance. Subsequent to orthogonal partial least squares-discriminant analysis, 156 metabolites displaying significant intergroup differences underwent identification. These differential metabolites mainly consisted of amino acids, lipids, nucleotides, and metabolites associated with energy metabolism. The details of all 156 differential metabolites identified by OPLS-DA, including metabolite names, annotation levels (HMDB/METLIN/LipidMaps), VIP scores, fold changes, adjusted *P* values, and pathway classifications, were shown in Supplementary Table 1.

Among plasma metabolites, levels of leucine, PC(16:0/20:4), and malic acid showed a progressive decline extending from control cohort toward MCI cohort, subsequently advancing toward AD cohort, with statistically significant intergroup differences observed [leucine: (109.55 ± 18.25) μmol/L, (104.91 ± 20.61) μmol/L, and (90.91 ± 25.01) μmol/L, respectively, *F* = 17.135, *P* < 0.001; PC(16:0/20:4): 135.65 ± 25.75, 128.35 ± 28.85, and 109.35 ± 33.45 relative intensity × 104, respectively, *F* = 17.761, *P* < 0.001; malic acid: [(8.24 ± 2.41) μmol/L, (7.52 ± 2.68) μmol/L, and (7.35 ± 2.33) μmol/L, respectively, *F* = 3.044, *P* = 0.049)]. In terms of amino acid metabolism, branched-chain amino acids including isoleucine and valine were also significantly decreased in the AD group compared with the control group (see Table 4). Conversely, homocysteine, ceramide d18:1/24:1, ceramide/sphingomyelin ratio, and GSSG/GSH ratio exhibited a stepwise increase across the three groups [homocysteine: (9.85 ± 3.15) μmol/L, (10.45 ± 3.85) μmol/L, and (11.45 ± 4.42) μmol/L, respectively, *F* = 3.716, *P* = 0.026; ceramide d18:1/24:1: 19.65 ± 4.75, 24.65 ± 7.45, and 26.25 ± 7.85 relative intensity × 10^4^, respectively, *F* = 21.263, *P* < 0.001; ceramide/sphingomyelin ratio: median (interquartile range) values of 0.09 (0.07, 0.12), 0.11 (0.08, 0.14), and 0.11 (0.08, 0.16), respectively, *F* = 3.310, *P* = 0.038; GSSG/GSH ratio: 0.08 (0.06, 0.11), 0.09 (0.06, 0.12), and 0.10 (0.07, 0.13), respectively, *F* = 4.062, *P* = 0.018]. No statistically significant intergroup differences emerged regarding remaining metabolites among three groups (*P* > 0.05).

**TABLE 4:** Comparison of differential plasma metabolites among the three groups.

Metabolite	Control group (*n* = 83)	MCI group (*n* = 82)	AD group (*n* = 85)	*F-*value	*P-*value
Amino acids (μmol/L)					
Leucine	109.55 ± 18.25	104.91 ± 20.61	90.91 ± 25.01	17.135	<0.001
Isoleucine	63.15 ± 17.35	59.95 ± 16.85	57.65 ± 15.65	2.316	0.101
Valine	215.55 ± 62.65	204.75 ± 58.95	193.75 ± 50.35	3.020	0.051
Phenylalanine	62.05 ± 14.65	58.35 ± 13.25	56.85 ± 14.85	2.940	0.055
Tyrosine	68.35 ± 10.85	65.75 ± 12.35	63.85 ± 14.15	2.723	0.068
Glutamate	80.35 ± 17.15	76.75 ± 18.65	74.35 ± 17.85	2.389	0.094
Glutamine	502.65 ± 87.75	489.35 ± 89.35	469.55 ± 91.55	2.917	0.056
Homocysteine	9.85 ± 3.15	10.45 ± 3.85	11.45 ± 4.42	3.716	0.026
Lipids (relative intensity × 104)					
PC(16:0/18:1)	170.35 ± 58.35	156.75 ± 54.55	151.15 ± 59.75	2.456	0.088
PC(16:0/20:4)	135.65 ± 25.75	128.35 ± 28.85	109.35 ± 33.45	17.761	<0.001
PC(18:0/20:4)	98.65 ± 26.25	92.35 ± 25.15	89.35 ± 25.65	2.858	0.059
Sphingomyelin d18:1/16:0	257.35 ± 68.85	241.75 ± 66.35	233.75 ± 64.65	2.718	0.068
Sphingomyelin d18:1/24:1	181.45 ± 65.25	172.85 ± 67.75	157.35 ± 64.85	2.886	0.058
Ceramide d18:1/16:0	24.75 ± 7.55	26.45 ± 7.15	27.05 ± 7.84	2.101	0.125
Ceramide d18:1/24:1	19.65 ± 4.75	24.65 ± 7.45	26.25 ± 7.85	21.263	<0.001
Ceramide/sphingomyelin ratio	[0.09(0.07, 0.12)]	[0.11(0.08, 0.14)]	[0.11(0.08, 0.16)]	3.310	0.038
Energy metabolites (μmol/L)					
Citrate	102.45 ± 22.65	97.35 ± 20.75	94.45 ± 24.15	2.695	0.070
Succinate	4.52 ± 1.28	4.23 ± 1.21	4.08 ± 1.24	2.741	0.066
Malic acid	8.24 ± 2.41	7.52 ± 2.68	7.35 ± 2.33	3.044	0.049
Oxidative stress markers (μmol/L)					
Oxidized glutathione	3.19 ± 0.94	3.46 ± 1.18	3.61 ± 1.27	2.911	0.056
Reduced glutathione	40.04 ± 9.75	38.25 ± 9.95	36.95 ± 10.35	2.024	0.134
GSSG/GSH ratio	[0.08(0.06, 0.11)]	[0.09(0.06, 0.12)]	[0.10(0.07, 0.13)]	4.062	0.018
Malondialdehyde	2.56 ± 0.97	2.72 ± 0.93	2.90 ± 0.91	2.790	0.063

### Correlation analysis between retinal parameters and plasma metabolites

**FIGURE 2:**
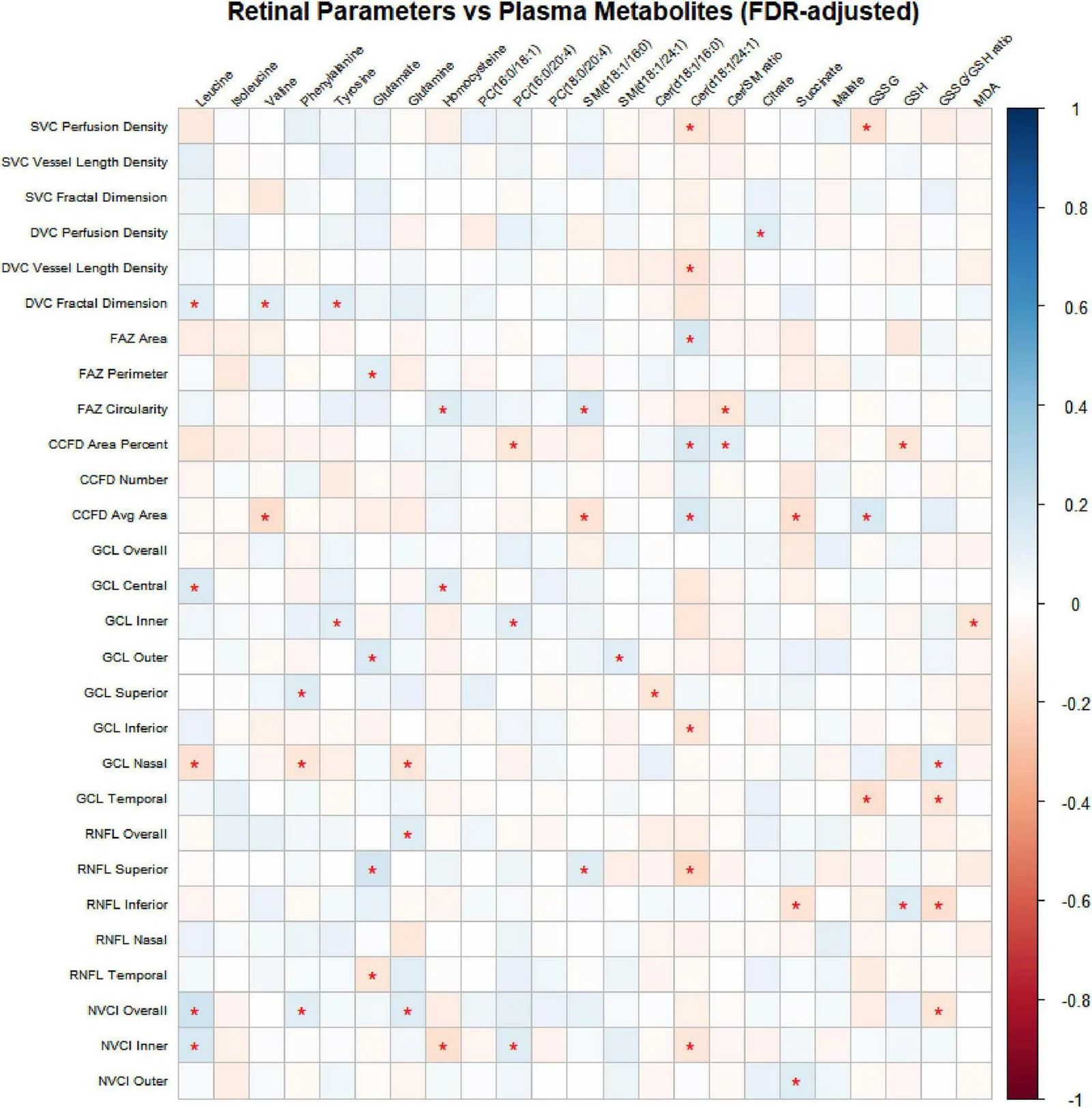
Heatmap of correlations between retinal parameters and plasma metabolites. *FDR-adjusted *q* < 0.05, marking statistically significant results.

To further explore associations linking retinal neurovascular alterations as well as systemic metabolic dysregulation, Spearman correlation analyses were performed between major retinal parameters and plasma metabolites. The results, as illustrated in Figure 2, demonstrated that NVCI was significantly positively correlated with plasma branched-chain amino acid levels. Specifically, overall NVCI was positively correlated with leucine levels (*r* = 0.205, *q* = 0.0011), while inner-ring NVCI also exhibited a positive correlation with leucine (*r* = 0.167, *q* = 0.0080). In contrast, NVCI showed a significant negative correlation with ceramide levels, and inner-ring NVCI was negatively correlated with ceramide d18:1/24:1 (*r* = −0.139, *q* = 0.0280). Perfusion density involving deep vascular plexus displayed positive associations with tricarboxylic acid cycle intermediates, particularly citrate (*r* = 0.145, *q* = 0.0219). Percentage area regarding CCFDs exhibited correlations involving oxidative stress markers, presenting inverse association with reduced glutathione (*r* = −0.132, *q* = 0.0377), whereas mean CCFDs area demonstrated positive association involving oxidized glutathione (*r* = 0.165, *q* = 0.0091). Additionally, FAZ circularity index displayed positive association with homocysteine concentration (*r* = 0.146, *q* = 0.0211). These correlations, while statistically significant, were modest in magnitude, suggesting exploratory associations that may reflect shared pathological processes rather than direct causal relationships.

### Establishment and validation of the integrated diagnostic model

To evaluate the diagnostic value of retinal neurovascular parameters combined with plasma metabolic biomarkers for the early detection of AD, multivariate stepwise regression analysis underwent performance to determine independent predictive factors, followed by establishment of an integrated diagnostic model. In the stepwise regression model, age, sex, education, BMI, systolic blood pressure, diastolic blood pressure, and fasting blood glucose were entered as candidate variables; non-significant confounders were excluded during model selection, with the final retained predictors shown in Table 5. Among the retinal parameters included in the analysis, FAZ area, percentage area of CCFDs, and NVCI were retained in the final model. Among plasma metabolites, leucine, ceramide d18:1/24:1, and PC(16:0/20:4) were identified as independent predictive factors.

**TABLE 5:** Multivariate stepwise regression analysis for the diagnosis of AD.

Variable	Regression coefficient	Standard error	Wald χ ^2^ value	*P*-value	OR value	95% CI
FAZ area	4.656	2.026	5.284	0.022	105.230	1.986–5576.131
Percentage of CCFDs	0.160	0.046	11.874	0.001	1.173	1.071–1.285
NVCI	−2.768	0.732	14.284	<0.001	0.063	0.015–0.264
Leucine	−0.023	0.008	8.285	0.004	0.977	0.962–0.993
Ceramide d18:1/24:1	0.066	0.024	7.459	0.006	1.068	1.019–1.120
PC(16:0/20:4)	−0.023	0.006	15.095	<0.001	0.977	0.966–0.989
Constant	3.203	1.731	3.426	0.064	24.613	–

**FIGURE 3:**
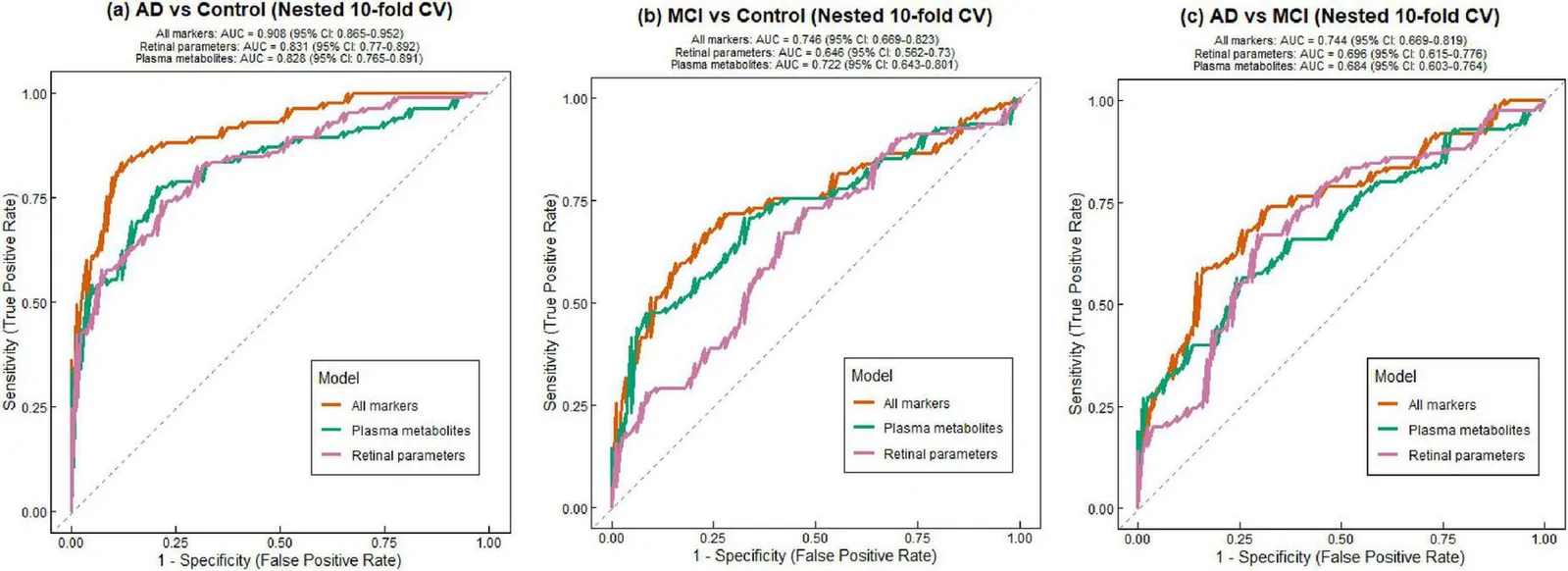
Model performance validation. **(A)** ROC curve of the integrated model for distinguishing AD from cognitively normal controls. **(B)** ROC curve of the integrated model for distinguishing MCI from cognitively normal controls. **(C)** ROC curves of the integrated model for distinguishing AD from MCI.

The integrated diagnostic model based on the six independent predictive factors exhibited enhanced diagnostic performance relative to single-parameter models, as illustrated in Figure 3. Following nested 10-fold cross-validation, the model achieved an AUC of 0.908 for differentiating AD cases from cognitively healthy controls, with a 95% confidence interval ranging from 0.865 to 0.952. Under optimal cutoff threshold, sensitivity and specificity reached 83.5 and 88.0%, respectively. In comparison, AUCs achieved using retinal parameters alone and plasma metabolic biomarkers alone were 0.831 and 0.828, respectively, indicating that the integrated model significantly outperformed either single-modality approach. For the diagnosis of MCI, the integrated model yielded an AUC of 0.746, with a 95% confidence interval of 0.669–0.823, a sensitivity of 72.0, and a specificity of 72.3%. Furthermore, the AUC for discriminating MCI from AD was 0.744, with a 95% confidence interval ranging from 0.669 to 0.819, a sensitivity of 57.6, and a specificity of 85.4%.

**FIGURE 4:**
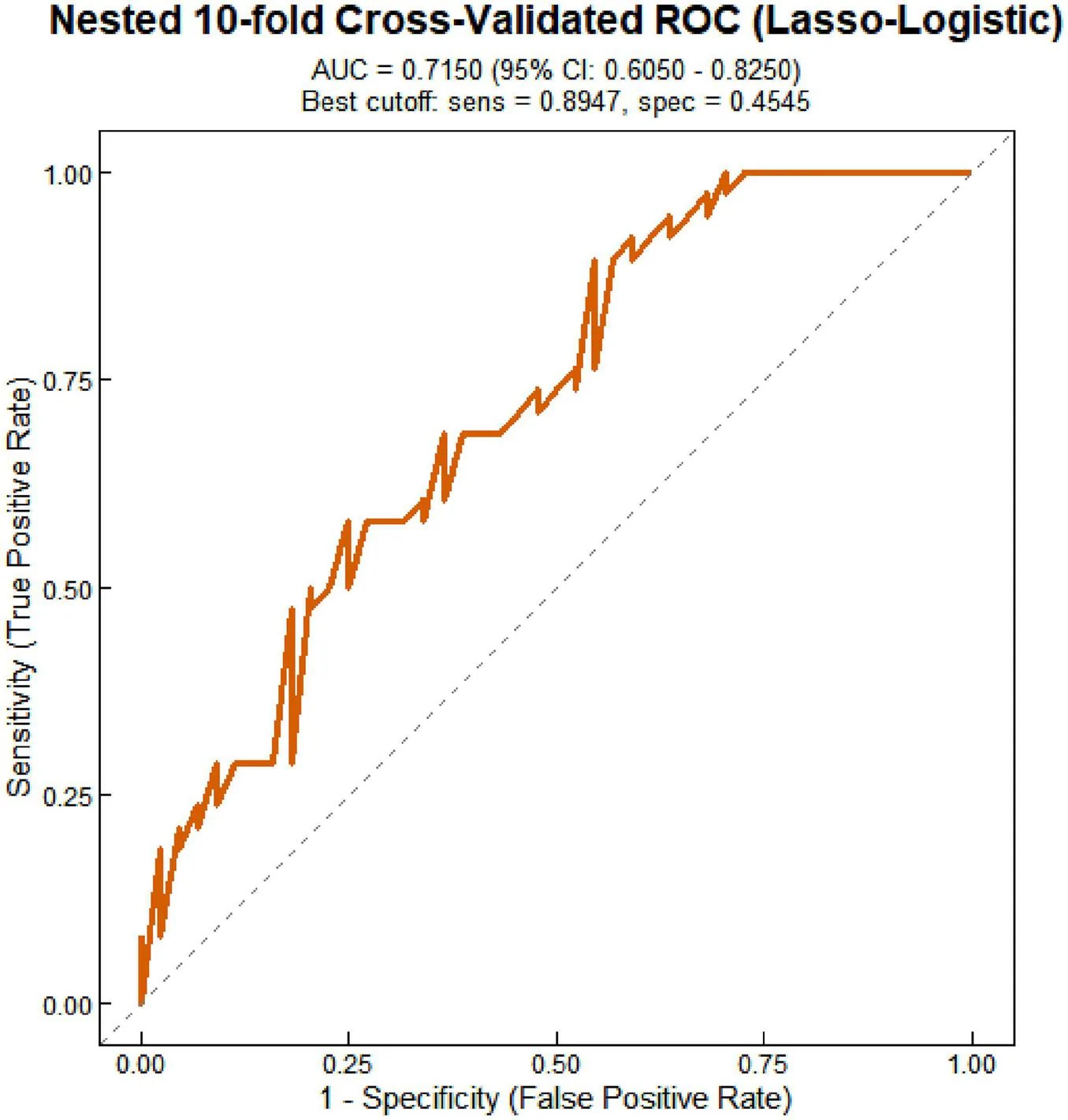
ROC curve of the integrated model for predicting conversion from MCI to AD.

Additional analyses were performed to evaluate the predictive utility of the integrated model for progression from MCI to AD. Eighty-two MCI patients were stratified into high-risk and low-risk cohorts according to baseline integrated model scores. During a mean follow-up duration of (21.13 ± 2.93) months, 30 of 47 patients in the high-risk cohort progressed to AD, corresponding to a conversion rate of 63.83%, whereas only 8 of 35 patients in the low-risk group developed AD, corresponding to a conversion rate of 22.86%. The difference in conversion rates between the two cohorts was statistically significant. The integrated model yielded an AUC of 0.715 for predicting progression from MCI to AD, with a 95% confidence interval spanning 0.605–0.825, a sensitivity of 89.47%, and a specificity of 45.45%, indicating potential clinical applicability regarding early-stage risk stratification associated with AD. Corresponding findings are shown in Figure 4.

### Subgroup analysis

To further explore effects associated with distinct clinical characteristics upon retinal neurovascular alterations alongside metabolic dysregulation, subgroup analyses were conducted according to age, sex, alongside disease severity. Findings appear summarized throughout Tables 6–8. No statistically significant differences emerged between subgroup comparisons regarding NVCI, FAZ area, CCFDs, leucine, ceramide d18:1/24:1, or PC(16:0/20:4) (*P* > 0.05).

**TABLE 6:** Comparison of major parameters among different subgroups of patients with AD.

Age subgroup	≥ 75 years group (*n* = 23)	< 75 years group (*n* = 62)	*t*-value	*P-*value
NVCI	1.41 ± 0.26	1.45 ± 0.24	−0.850	0.398
FAZ area (mm^2^)	[0.36(0.31, 0.44)]	[0.36(0.32, 0.39)]	−0.352	0.726
CCFDs (%)	16.11 ± 4.63	15.70 ± 4.51	0.386	0.700
Leucine (μmol/L)	91.71 ± 28.81	90.57 ± 23.72	0.192	0.848
Ceramide d18:1/24:1	25.14 ± 8.86	26.74 ± 7.39	−0.862	0.391
PC(16:0/20:4)	109.93 ± 31.87	109.10 ± 34.89	0.105	0.917

**TABLE 7:** Sex subgroup continued table.

Sex subgroup	Male group (*n* = 23)	Female group (*n* = 62)	*t-*value	*P-*value
NVCI	1.46 ± 0.29	1.41 ± 0.21	0.913	0.364
FAZ area (mm^2^)	[0.35(0.31, 0.42)]	[0.36(0.31, 0.43)]	−0.078	0.938
CCFDs (%)	16.50 ± 4.25	15.14 ± 4.74	1.391	0.168
Leucine (μmol/L)	92.81 ± 25.08	88.98 ± 25.08	0.704	0.484
Ceramide d18:1/24:1	27.32 ± 6.05	25.15 ± 9.28	1.281	0.204
PC(16:0/20:4)	115.36 ± 32.29	103.19 ± 33.87	1.697	0.093

**TABLE 8:** Disease severity subgroup continued table.

Disease severity subgroup	Mild group (MMSE 20–24) (*n* = 23)	Moderate group (MMSE < 20) (*n* = 62)	*t*-value	*P*-value
NVCI	1.48 ± 0.25	1.39 ± 0.23	−1.706	0.092
FAZ area (mm^2^)	[0.35(0.31, 0.42)]	[0.37(0.28, 0.44)]	0.250	0.803
CCFDs (%)	15.15 ± 4.09	16.46 ± 4.86	1.343	0.183
Leucine (μmol/L)	93.34 ± 25.92	88.65 ± 24.20	−0.864	0.390
Ceramide d18:1/24:1	26.62 ± 7.94	25.91 ± 7.84	−0.417	0.678
PC(16:0/20:4)	108.78 ± 38.07	109.88 ± 28.93	0.150	0.881

### Discussion

This research systematically evaluated characteristics and interrelationships of retinal neurovascular alterations and plasma metabolic dysregulation in patients with AD through the combined application of OCTA and metabolomic analyses, thereby providing novel insights into the early pathological mechanisms underlying AD. The findings demonstrated that patients with AD exhibited significant reductions in retinal vascular perfusion density, thinning of the ganglion cell layer, and decreased NVCI, accompanied by multiple metabolic abnormalities, including reduced branched-chain amino acid levels and elevated ceramide concentrations. Importantly, these alterations were already detectable during the MCI stage, suggesting that dysfunction of the neurovascular unit and metabolic imbalance may be important early features associated with AD.

Findings derived throughout current study demonstrated that superficial vascular plexus perfusion density within AD cohort declined by 7.1% relative toward control cohort (*P* < 0.001), whereas FAZ area was enlarged by 16.1% (*P* < 0.001), CCFDs increased by 28.3% (*P* < 0.001), ganglion cell layer thickness decreased by 18.3% (*P* < 0.001), and overall NVCI declined by 12.7% (*P* < 0.001). Similar parameters in the MCI group exhibited a stepwise pattern of alteration, indicating that dysfunction of the retinal neurovascular unit may already occur during the early stages of AD (Chen et al., 2025; Wang et al., 2021). Plasma metabolomic analysis further revealed that leucine levels were reduced by 17.0% (*P* < 0.001), PC(16:0/20:4) levels were decreased by 19.4% (*P* < 0.001), ceramide d18:1/24:1 levels were elevated by 33.6% (*P* < 0.001), and the ceramide/sphingomyelin ratio increased by 22.2% (*P* < 0.001) in the AD group. Correlation analyses revealed modest but statistically significant associations between NVCI and leucine (*r* = 0.205, *q* = 0.0011) and between NVCI and ceramide d18:1/24:1 (*r* = −0.139, *q* = 0.0280). While these correlations suggest potential interconnections between retinal neurovascular coupling and systemic metabolic status, the effect sizes are small and should be interpreted cautiously. These findings likely reflect indirect links mediated by shared pathological mechanisms—such as neurovascular unit dysfunction, mitochondrial stress, and neuroinflammation—rather than direct biological causality, and require validation in independent, longitudinal cohorts. Moreover, integrated diagnostic model incorporating FAZ area, CCFDs, NVCI, leucine, ceramide d18:1/24:1, and PC(16:0/20:4) achieved an AUC reaching 0.908 (95% CI: 0.865–0.952) regarding differentiation between AD cases alongside controls, accompanied through sensitivity reaching 83.5% together with specificity reaching 88.0%. Additionally, model performance yielded an AUC reaching 0.715 (95% CI: 0.605–0.825) regarding prediction involving progression from MCI toward AD.

Reductions in retinal vascular density, enlargement of the FAZ, increased CCFDs, ganglion cell layer thinning, and significant decreases in NVCI were observed in patients with AD, suggesting an imbalance between neuronal metabolic demand and local blood supply. Chen et al. (2025) proposed that neurovascular dysfunction may represent one of the earliest reliable biomarkers of AD. In the present study, NVCI in patients with AD was reduced by 12.7% relative to controls, indicating that this abnormality may already be detectable during the MCI stage. The mechanisms underlying neurovascular coupling dysfunction are believed to involve Aβ deposition-induced pericyte loss and vascular inflammatory responses, which impair blood–retinal barrier integrity and weaken vasodilatory capacity (Kisler et al., 2017; Sweeney et al., 2016). The observed increase in CCFDs further suggested the presence of hypoxia and metabolic stress within the outer retina, consistent alongside findings reported by Wang et al. (2021) regarding decreased retinal vascular density among AD patients. A recent review by Min et al. further emphasized that early microvascular defects detected by OCTA may reflect the pathological basis of neurovascular dysfunction in AD (Min et al., 2025).

Associations linking decreased plasma branched-chain amino acids alongside elevated AD risk received additional validation throughout current study. Leucine concentrations within AD cohort declined by 17.0% and demonstrated a positive correlation with NVCI. A large prospective investigation performed through Tynkkynen et al. (2018) demonstrated that decreased plasma branched-chain amino acid concentrations correlated with increased risks involving dementia together with AD, whereas Ikeuchi et al. (2022) observed similar findings in individuals with MCI. It should be noted that several previous studies have reported elevated branched-chain amino acid levels in patients with AD (Siddik et al., 2022). Such discrepancies may be attributable to differences in metabolic status, comorbidities, particularly type 2 diabetes mellitus, and disease stage among study populations. Deficiency of branched-chain amino acids may accelerate neurodegeneration through restriction of protein synthesis, disruption of the glutamate–glutamine cycle, and impairment of mitochondrial energy metabolism (Sato et al., 2021). In contrast, ceramide accumulation exerts multiple toxic effects on the neurovascular unit through promotion of Aβ generation, induction of apoptosis, and aggravation of neuroinflammation (Jazvinšcak Jembrek et al., 2015; Mielke and Haughey, 2012). In the present study, ceramide d18:1/24:1 levels in the AD group were elevated by 33.6%, whereas the ceramide/sphingomyelin ratio increased by 22.2%, findings that were highly consistent with previous multi-omics investigations described through Baloni et al. (2022) alongside Uranbileg et al. (2024). Inverse association identified involving ceramides and NVCI further suggested that ceramides may directly impair endothelial function, disrupt blood–brain barrier integrity, and attenuate vasodilatory responses (Vance and Vance, 2009). The integrated diagnostic model combining retinal parameters and plasma metabolites achieved an AUC of 0.908, which was markedly superior to models based on retinal parameters alone (AUC = 0.831) or metabolites alone (AUC = 0.828). These findings suggested that integration of multidimensional information reflecting both local neurovascular alterations and systemic metabolic status may provide a more comprehensive representation of the complex pathological processes underlying AD. Furthermore, model yielded an AUC reaching 0.715 regarding prediction involving progression from MCI to AD, indicating its potential utility as a noninvasive and reproducible tool for early risk stratification (Perron et al., 2025).

Several limitations of the current study should be acknowledged. First, the cross-sectional design prevented determination of causal associations. Second, the study was conducted in a single-center setting with a relatively limited sample size, and external validation remains necessary. Third, other retinal microvascular networks, such as radial peripapillary capillaries, were not included in the analysis. Fourth, we acknowledge that our diagnostic classification was primarily based on clinical criteria, cognitive assessments, and MRI findings, and did not include biomarker confirmation using amyloid PET, tau PET, or CSF biomarkers (ATN framework). This approach reflects the diagnostic standards applicable at the time of study recruitment. However, we recognize that the recently updated NIA-AA 2024 criteria emphasize a biological definition of AD, which may influence the generalizability of our findings. Future studies incorporating these validated biomarkers are warranted to confirm the association of our integrated model with the core pathological hallmarks of AD. Fifth, we acknowledge that the MCI-to-AD progression analysis was limited by the relatively small number of progression events (38 of 82 MCI patients, 46.3%) during follow-up. This limited event count precluded the use of multivariable Cox proportional hazards regression, as the inclusion of multiple predictors would carry a high risk of model overfitting and unstable effect estimates. Therefore, we adopted high-risk versus low-risk stratification as an alternative approach to compare progression rates between groups, and the results showed a significant difference (63.83% vs. 22.86%). Future large-scale, multicenter prospective studies with longer follow-up durations and larger numbers of conversion events are warranted to validate our findings using Cox regression and to establish more robust predictive models for MCI-to-AD progression. Sixth, although we carefully controlled for major demographic and clinical confounders (age, sex, education, BMI, blood pressure, and glucose) in our regression models, we acknowledge that several other factors—such as axial length, intraocular pressure, lens status, medication use (antihypertensives, lipid-lowering agents), renal function, and dietary habits—were not assessed in the current study. These variables may potentially influence retinal vascular parameters or plasma metabolite levels and thus represent unmeasured confounders. However, the consistency of our findings across subgroup analyses (by age, sex, and disease severity) and the robustness of the integrated model after adjusting for major confounders suggest that the observed associations are unlikely to be substantially driven by these unmeasured factors. Future studies with more comprehensive phenotyping are warranted to further validate our results. Finally, although participants with overt ocular diseases were excluded, the potential influence of subclinical vascular abnormalities could not be completely eliminated. Future longitudinal investigations are warranted to dynamically monitor temporal changes in biomarkers and to integrate multimodal analyses incorporating neuroimaging findings alongside cerebrospinal fluid biomarkers. Additionally, integration involving artificial intelligence together with deep learning technologies may further improve the automation and diagnostic accuracy of retinal image analysis, thereby facilitating the clinical translation of retinal biomarkers in AD (Wisely et al., 2022).

In conclusion, the present study systematically characterized retinal neurovascular coupling impairment among AD patients alongside elucidation regarding intrinsic associations involving plasma metabolic dysregulation. The findings revealed the significant associations of neurovascular unit dysfunction, branched-chain amino acid deficiency, ceramide accumulation, and disturbances in energy metabolism and redox homeostasis with AD. The integration of retinal neurovascular parameters with plasma metabolic biomarkers may provide a valuable approach regarding early-stage diagnosis together with risk assessment of AD and demonstrates promising potential for future clinical application.

## Data Availability

The original contributions presented in the study are included in the supplementary material, further inquiries can be directed to the corresponding authors.
